# Inline Skating as an Additional Activity for Alpine Skiing: The Role of the Outside Leg in Short Turn Performance

**DOI:** 10.3390/ijerph19031747

**Published:** 2022-02-03

**Authors:** Vjekoslav Cigrovski, Mateja Očić, Ivan Bon, Branka Matković, Peter Šagát

**Affiliations:** 1 Laboratory for Sports Games, Faculty of Kinesiology, University of Zagreb, 10000 Zagreb, Croatia; vjekoslav.cigrovski@kif.unizg.hr (V.C.); mateja.ocic@kif.unizg.hr (M.O.); branka.matkovic@kif.unizg.hr (B.M.); 2 Health and Physical Education Department, Prince Sultan University Riyadh, Riyadh 66833, Saudi Arabia; sagat@seznam.cz

**Keywords:** biomechanical analysis, pressure insoles, Xsens motion capture system, performance analysis, recreational skiers, dry-land training

## Abstract

The complexity of skiing movements urges recreational alpine skiers and competitors to undertake many specific skill trainings not only during the season but also during the off-season using alternative sports. In AS, the role of the outside leg is crucial for successful turn performance. By measuring kinematic and kinetic parameters, we could define whether there is an objective similarity of the role and the movements of the outside leg while performing a turn in AS to those in the most used additional activity, IS. The sample consisted of ten female alpine ski instructors (age 31.6 ± 8.23, height 170.66 ± 7.32 cm, weight 60.16 ± 7.58 kg). Overall, 280 turns were analyzed (140 for AS and 140 for IS). For the purposes of this study, the variable sample consisted of 14 variables in total. For the detection of differences between short turn performance in AS and IS, MANOVA was used. The main findings of our study are defined similarities in pressure distribution during IS and AS and noticeable differences in the kinematic parameters of the outside leg between the mentioned activities. Based on the gathered results, recreational alpine skiers should be aware that IS cannot be used for the purpose of AS adoption, but rather as a dry-land additional activity for AS preparation.

## 1. Introduction

Alpine skiing (AS) is a specific activity consisting of complex and uncommon movements of the human body [1]. Specific movements that a skier performs are then transferred to ski boots, skis and consequently the snow surface. Finally, the ground reaction force (GRF) then enables the alpine skier to steer the skis and complete turns [2]. In order to control the speed and direction during a descent, the skier has to maintain the optimal posture by timely shifts in the center of their mass, optimally applying pressure and utilizing GRF [3].

In addition, the edging angle is another important factor that secures the skier’s reliable support on the snow surface. If all of the abovementioned factors are considered, each phase of the turn will be successfully performed without skidding and sliding [4,5,6].

The outside leg is more exposed to skidding when compared to the inside leg when the steepness of the terrain and applied pressure on both legs are the same. Therefore, the role of the outside leg is especially important in the conditions of very steep and icy terrain. That is the main reason for shifting the pressure predominantly on the outside leg, which is placed further from the projection of the center of mass [7]. In order to be successful in applying pressure on the outside ski, the skier must be in a specific body position, especially in the finishing phases of a ski turn. This position requires hip and knee flexion, and also a side arc that is achieved by moving the upper and lower leg in the direction of the center of the turn and the upper body in the opposite direction [8,9,10].

The complexity of the skiing movements urges alpine ski competitors to perform a large number of specific skills trainings not only during the season but also during the off-season using alternative sports. Those must include similar body positions, relations between body segments, coordination structures, general movements and muscle contractions when compared to the primary activity [11].

One of the most frequently used alternative activity for physical and technical preparation for AS is inline skating (IS) [12]. IS enables the execution of short parallel turns that are similar to skiing turns [13,14]. Even though there are clear similarities between the two mentioned activities, the surface, equipment and factors related to both are different [11]. IS is usually performed on a less steep terrain, which is also more solid (concrete or asphalt). Furthermore, during the turn performance in IS, the contact with the terrain surface is made through wheels placed exactly beneath the feet’s centers. In AS, on the other hand, the contact is made by the ski edge, which is more lateral. In addition, the speed also varies, which can affect relations between body segments and, finally, forces (GRF and centrifugal force), when observing the same moment of the turn performance in both activities [15].

Comparing the turn execution on roller skates and skis, it can be assumed that alpine skiers can benefit in terms of technical training and physical conditioning by using IS as an additional activity [16]. Research conducted by Kroll et al. [11] suggests a similar sequence of body movements during IS and AS.

In AS, as already mentioned, the role of the outside leg is considered to be crucial in successful turn performance. The inside leg has the role of a support, while the outside leg mostly controls the direction, duration and speed, and also prevents the skier from skidding by adjusting the edging angle in the turn [9]. Research regarding movement analysis in IS is scarce so it is not clear whether the described movement patterns and roles of the inside and the outside legs are the same while executing parallel turns in IS.

To objectively identify and compare the roles of the outside leg in IS and AS, it is necessary to determine the joint angles and pressure forces that occur while performing a turn.

Combined kinematic and kinetic analysis can provide objective evaluation of the outside leg movements and pressure distributions while skating and skiing. By measuring kinematic and kinetic parameters in this research, we could define whether there is an objective similarity of the role and the movements of the outside leg while performing a turn in IS and AS. The results gained emphasize the function of IS as an additional activity for developing and maintaining technical and physical conditioning outside the skiing season for competitive but also recreational level alpine skiers, who do not have the opportunity to spend a great amount of time on the ski slopes. Therefore, alternative activities are even more necessary to prepare them for their short skiing season.

We hypothesized that the short turn execution during AS induces a higher pressure on the outside foot than it does in IS due to the slope steepness. Regarding the pressure distribution, we assumed there would be no significant differences in the load distribution during the turn execution in both activities. Furthermore, we assumed there would be similarities in the kinematic parameters of the lower limbs during short turn performance in IS and AS.

## 2. Materials and Methods

Participants: The sample consisted of ten female alpine ski instructors (age 31.6 ± 8.23, height 170.66 ± 7.32 cm, weight 60.16 ± 7.58 kg). They did not report any prior injuries that could affect their AS and IS technique or kinetic and kinematic variables while performing turns in both activities. All participants had previous experience in inline skating and had finished basic inline skating school. Participants gave their written consent to participate in this study after being informed in detail about the aims and protocol of the research. The Faculty of Kinesiology, University of Zagreb (Croatia) Ethics Committee approved the study, which was performed following the ethical standards of the Declaration of Helsinki.

Variables: The mentioned variables were selected based on relevant studies focused on the kinematic and kinetic analysis of AS technique [6,8,9,10,17,18,19,20,21]. Based on those studies, the determinants of a successfully executed turn are a proper pressure distribution of certain foot regions in specific phases of the turn and a proper edging angle that requires optimal relations between lower body segments. Considering the fact that lower extremities have a key role in performing a successful turn—especially when observing the outside leg—for the purpose of the kinematic analysis the focus was on parameters that describe the movements of the outside leg. The main movements that enable a skier to steer their skis are flexion and extension (up–down movements), abduction and adduction (lateral movements) and rotations [4]. Our research focused on variables that represent the amplitude of movements in some joints of the lower extremities (knee and hip), with emphasis on flexion–extension and abduction–adduction. Kinetic variables refer to the pressure of the outside leg during the execution of turns. All the available regions of foot pressure enabled by the manufacturer were analyzed. This furthermore enabled the observation of the pressure of the heel and the lateral and medial parts of the foot throughout the turn. The skis are steered throughout the turn mainly by moving the lower extremities. Because of the already mentioned role of the outside leg as a crucial factor in optimal turn execution, it can be assumed that the potential differences or similarities between observed activities would appear in the analysis of the outside leg parameters.

Analyses of kinetic and kinematic parameters were conducted on short parallel turns in two activities—AS and IS. Overall, 280 turns were analyzed (140 for AS and 140 for IS). For the purposes of this study, the variable sample consisted of 14 variables in total. The following kinetic variables were measured: the maximum force of the right foot in the left turn (Max_R_LT); the force of the lateral side of the right foot in the left turn (Lat_R_LT); the force of the medial side of the right foot in the left turn (Med_R_LT); the force of the right heel in the left turn (He_R_LT); the maximum force of the left foot in the right turn (Max_L_RT); the force of the lateral side of the left foot in the right turn (Lat_L_RT); the force of the medial side of the left foot in the right turn (Med_L_RT); and the force of the left heel in the right turn (HE_F_L_RT). All results are shown in newtons (N). Pressure data are also presented in terms of mean body weight (MBW= pressure (kg)/mean body weight of all participants (kg)).

The following kinematic variables were measured (180° = knee and hip fully extended): the angle of the right knee flexion in the left turn (R_KNEE_LT); the angle of the right hip flexion in the left turn (R_HIP_F_LT); the angle of the right hip abduction in the left turn (R_HIP_AB_LT); the angle of the left knee flexion in the right turn (L_KNEE_RT); the angle of the left hip flexion in the right turn (L_HIP_F_RT); and the angle of the left hip abduction in the right turn (L_HIP_AB_RT). All results are shown in degrees (°).

**Figure 1:**
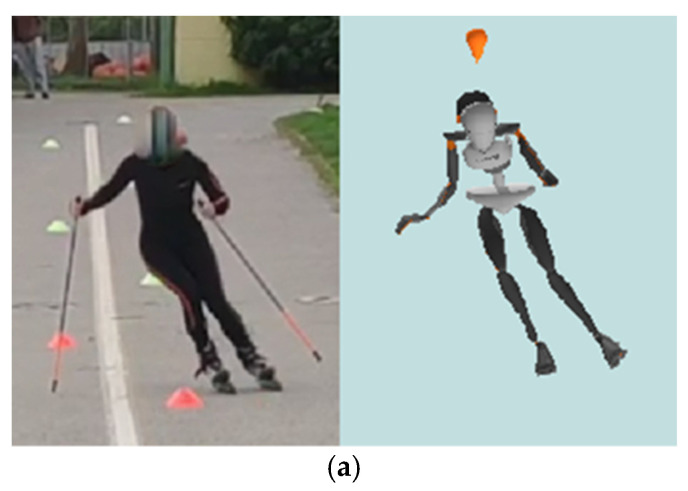

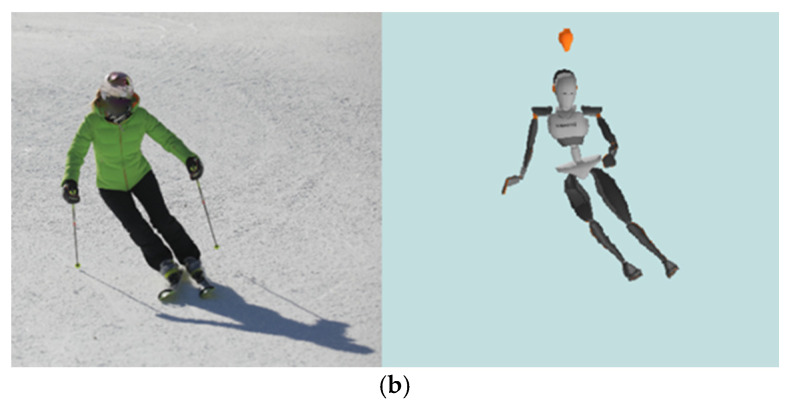
(**a**) Preview of IS turn; (**b**) preview of AS turn.

All data were obtained at the moment when participants’ feet were in a position parallel to the fall line (Figure 1). For the purposes of field testing, it is necessary to use systems and equipment that are not invasive and to secure accurate data without affecting the athlete’s performance [17,18,19,20,21].

The pressure distribution (kinetic variables) during AS and IS was measured with insoles designed for pressure detection (Novel, Pedar). Due to their construction (2 mm thin and very light), insoles had minimal influence on turn performance during testing of both dynamic activities. For the purposes of this study, output rate was set at 100 Hz and the data were derived from the corresponding software (Novel, Loadpad 25.3.6). The reliability and validity of Novel pressure insoles for analyzing pressure distribution were confirmed in previous studies of similar activities [2,11,22].

Kinematic parameters were measured by an Xsens MVN Link inertial suit system. The system consisted of 17 three-dimensional accelerometers/gyroscopes/magnetometers and a battery. The output rate was set at 240 Hz, which ensures real-time human motion analysis without affecting the movement or rate of motion. Data were derived from the corresponding MVN BIOMECH software (Xsens, MVN Studio 4.4, firmware version 4.3.1, Enschede, the Netherlands). Previous studies confirmed the reliability and validity of the Xsens kinematic suit for analyzing kinematic parameters (joint angles) in activities similar to those in this study [21,23].

Protocol of investigation: The protocol was the same for all participants and included measuring anthropometric characteristics, adjusting the Xsens suit and adjusting suitable pressure insoles. Anthropometric characteristics were used for adjusting the sensors and calibration of the Xsens system. The calibration was performed according to the standard of procedure advised by the manufacturer (Xsens technologies B.V., Netherlands). In addition, standard calibration of pressure insoles was performed according to the manufacturer’s advice (Novel GmbH, Munich, Germany). All turns were recorded with a video camera (Panasonic GH 5) for the purpose of synchronizing both kinetic and kinematic systems. In order to successfully synchronize the systems, participants were asked to alternately lift their skis or skates two times off the surface just before starting their descent. That was the starting moment when the time lapse of both systems was aligned.

Before testing procedures in AS, participants performed a free warm up run and a trial run in a defined corridor. Then, kinematic and kinetic parameters were measured with corresponding systems as participants performed short parallel turns in the defined corridor. The protocol of investigation was very similar to that used for the IS measurements. After adjusting kinematic and kinetic systems, participants had a free warm up run and then a trial run in the defined corridor. Afterwards, measurement of predefined kinematic and kinetic parameters was conducted while performing short parallel turns in the defined corridor. Participants received detailed instructions regarding short parallel turn performance both on skis and skates. Overall testing was conducted for four days; two days for measuring turns in AS and two days for measuring turns in IS. Testing procedures on the ski slope were performed in the morning hours to secure better snow conditions. The average incline of the slope was 20°. Participants had suitable skiing equipment (slalom skis and adjusted ski boots). Short parallel ski turns were performed in a 3 m wide corridor, according to protocols defined in alpine ski schools. Each turn had to be performed from one end of the corridor to another. Each participant performed 16 turns, but due to mistakes in motor movement (skidding, sliding, etc.), several turns were excluded from further analysis.

**Figure 2:**
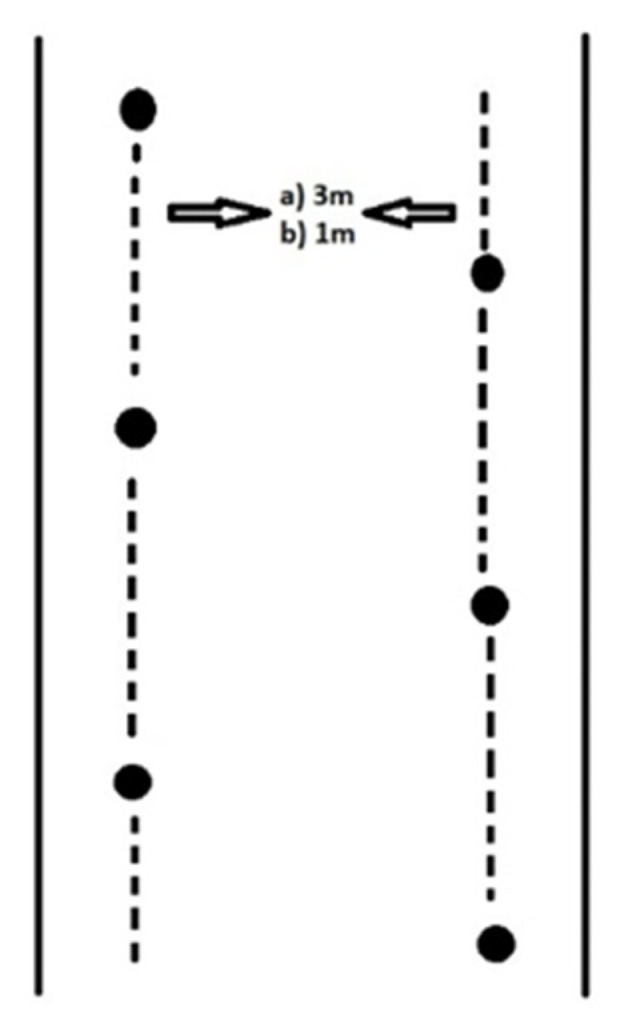
Preview of the corridor for short turn execution (**a**)—corridor width for AS; (**b**)—corridor width for IS).

Skating turns were performed in sunny weather conditions in order to secure a dry surface. The corridor width was 1 m and the average incline was 10°. Participants also performed 16 turns, but some of them were excluded from further analysis due to certain mistakes in motor movement. The defined corridor for both skiing and skating turns is presented in Figure 2.

Statistical analysis: Statistical package Statistica version 13.5.0.17 (TIBCO Software Inc., Palo Alto, CA, USA) was used for data analysis. Basic descriptive parameters for all measured variables were calculated. The normality of data distribution was tested by the Kolmogorov–Smirnov test. MANOVA was used for the detection of differences between turn performance in AS and IS. The results were considered significant when *p* < 0.05. With the use of the G*power program, the sample size (number of turns) was calculated (*n* = 122), which was needed for the measurement procedure with statistical significance of *p* < 0.05; statistical power 0.95; effect size 0.25; 2 groups.

## 3. Results

Differences in kinematic and kinetic parameters between IS and AS were tested by MANOVA; results are shown in Table 1.

Results shown in Table 1 show a statistically significant difference between observed short turns executed in IS and AS (F = 169.1; *p* < 0.01).

Basic descriptive parameters of each tested kinematic variable along with the results of MANOVA are presented in Table 2.

Table 2 shows differences in all observed kinematic variables between short turns executed during IS and AS (*p* < 0.01). In the left turn, the knee flexion differed significantly between IS and AS, with higher angle values detected in IS than it was in AS (157.43° compared to 139.56°). In addition, the flexion of the right hip was significantly different, with higher angle values determined while performing a turn in AS (145.01° compared to 139.03). Moreover, the hip abduction was significantly greater while performing a turn in AS than it was in IS (163.68° compared to 170.73°).

The results were the same when analyzing right turns. The knee flexion was greater while performing a short turn in AS compared to IS (140.30° compared to 159.93°). The hip was more flexed while performing a short turn in AS than it was in IS (138.33° compared to 145.39°). Regarding the hip abduction, it was determined to be greater during AS than it was in IS (163.00° compared to 170.51°).

Basic descriptive parameters of each tested kinetic variable, along with results of MANOVA, are presented in Table 3.

Table 3 shows significant differences between short turn performance in IS and AS in seven out of eight measured kinetic variables. In the left turn, pressure force values of the outside foot for AS are higher than those for IS for all observed variables, with the values of the heel pressure force being the only that did not differ significantly (*p* = 0.23). In relation to the MBW, the measured maximum force in AS was 1.27 MBW and in IS it was 0.97 MBW. Regarding the pressure distribution in both activities, the highest values of distribution were noted on the heel (IS—85.12% of maximal foot pressure; AS—70.06% of maximal foot pressure). The next highest proportions of pressure were located the on medial side of the foot (IS—9.15% of maximal foot pressure; AS—20.87% of maximal foot pressure). The lowest pressure values in the left turn in both activities were measured on the lateral side of the foot (IS—5.88% of maximal foot pressure; AS—9.07% of maximal foot pressure).

**Figure 3:**
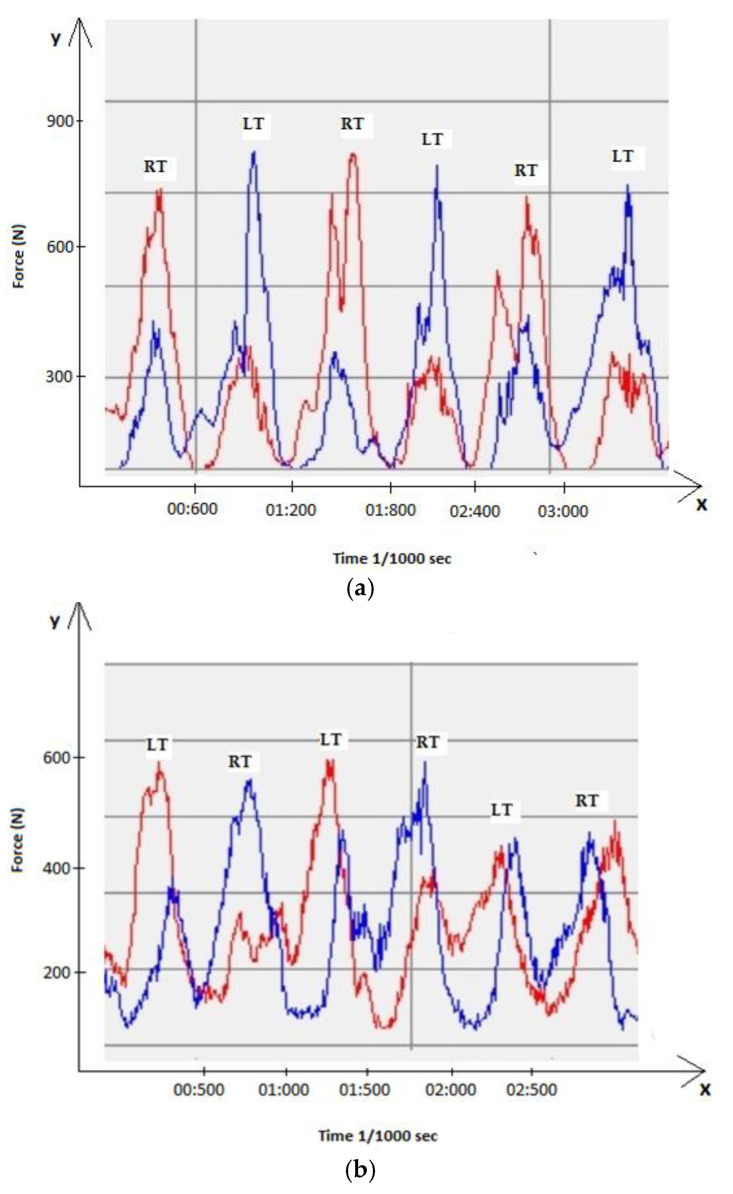
(**a**) Maximal pressure forces on left (red line) and right (blue line) foot during AS, LT—left turn, RT—right turn; (**b**) maximal pressure forces on left (red line) and right (blue line) foot during IS, LT—left turn, RT—right turn.

Furthermore, the measured forces of the outside foot in the right turn were also higher in AS than those in IS (Figure 3). In relation to the MBW, the measured maximum forces in AS and IS were 1.35 MBW and 0.94 MBW, respectively. In both turns, i.e., in IS and AS, the biggest proportion of foot pressure was noticed on the heel. Concerning the pressure distribution in both IS and AS, the highest values were detected on the heel (IS—80.61% of maximal foot pressure; AS—68.34% of maximal foot pressure). The second-highest proportions of the overall pressure (IS—11.57%; AS—19.88%) were on the medial side of the foot. The lowest proportions of pressure were measured on lateral side of the foot (IS—6.84%; AS—9.53%).

## 4. Discussion

The aim of this study was to objectively determine and compare the roles of the outside leg while performing short turns in IS and AS. The main findings of our study are defined similarities in pressure distribution during IS and AS and noticeable differences in kinematic parameters of the outside leg between the mentioned activities. Those findings are not completely in accordance with previously stated hypotheses. Therefore, based on the results of this study, recreational alpine skier should be aware that IS cannot be used for the purpose of AS adoption but rather as a dry-land additional activity for preparation for AS.

There were statistically significant differences between IS and AS in almost all of the measured kinetic parameters (*p* < 0.01). The highest values of maximal foot pressure were noticed in AS in both turns (Max_R_LT-AS—764.79 N, IS—584.82 N; Max_L_RT-AS—811.37 N, IS—564.44 N). Also, when comparing the ratio of maximum foot pressure and MBW, higher values were detected in AS (left turn, AS—1.27 MBW, IS—0.97 MBW; right turn, AS—1.35 MBW, IS—0.94 MBW). The mentioned results can be explained by some objective differences between the two activities. The steepness of the terrain directly affects the speed and gravitational force influencing the skier. Compared to IS, AS is performed usually on steeper slopes which consequently leads to higher speeds and higher centrifugal forces. Therefore, the skier must apply a higher foot pressure to master the speed, control the direction and overcome the high centrifugal force during a turn. As already stated, to successfully perform the turn on a steeper terrain, the skier must apply pressure predominantly on the outside leg [24]. When performing a short turn in IS, the terrain is less steep compared to that in AS, meaning the speed is not as high as it is in AS. The mentioned factors are directly connected to lower values of the applied foot pressure in the outside leg when performing short turns in IS. Furthermore, the friction between the equipment and the surface in the activities differs. It is easier to control and overcome skidding while performing turns on ski slopes by carving edges into the surface. This results in a more stable support, which also leads to higher values of the pressure force on the outside leg.

Similar results regarding differences in MBW and maximal foot pressure of the outside leg between IS and AS were found in a study conducted by Ropret [14]. Maximal values of foot pressure on the outside leg in AS were higher, ~1.5 MBW (in our study they were ~1.3 MBW). In IS, the measured foot pressure was 1.08 MBW (in our study it was ~0.97 MBW). The author concluded that the speed, turning radius and size of the centrifugal force is smaller in IS, which affects a reduced load on the outside leg when compared to AS.

Skis are much longer compared to inline skates, and the length and construction of the specific equipment influences the contact with the surface and differs between IS and AS. Those factors produce slightly different body positions and stances during turns. Despite the differences in equipment and maximal pressure force values, the distributions on foot regions were similar for IS and AS in both turns. The highest values of foot pressure were detected on the heel, followed by the medial side of the foot, and the lowest pressure was detected on the lateral side of the foot.

Dynamic balance is an important factor which has a great impact in AS on the turn performance [25,26]. The same can be stated for IS. Since, as mentioned, a skier adjusts their balance and applies the optimal pressure inside the ski boots and roller blades in the specific phase of the turn. The abovementioned factor is especially evident when both AS and IS are executed in a predefined corridor with the same gate distance and offset. Therefore, although the maximal pressure is higher in AS, the relative pressure and pressure distribution are quite similar.

In addition, the pattern of shifting the center of mass and maintaining a dynamic balance was similar when executing short parallel turns in observed activities. This suggests a positive transfer from developing dynamic balance on skates to applying it when executing turns in AS [27].

In research conducted by Kroll et al. [11], there were similarities but also differences in turn performance between skates and skis. The authors concluded that the main differences are the product of the different speeds of movement, turn radii and centrifugal forces. Furthermore, based on their results, the maximal foot pressure force in AS is on the outside leg while there is no significant difference between the foot pressures on the inside and the outside leg when observing the turn performance in IS. Even though there are differences, the authors suggest using short parallel turns executed on skates as an additional way to develop AS technique.

Regarding kinematic parameters, even though the coordination of movements and the sequence of lower limb actions is very similar in AS and IS, there were statistically significant differences in all of the measured kinematic variables (*p* < 0.01). Differences between the observed activities were similar in both turns. While performing a turn in AS, it was noticed that the side arc (hip abduction) was greater than that in IS (R_HIP_AB_LT, AS—63.68°, IS—170.73°; L_HIP_AB_RT, AS—163.00°, IS—170.51°). Moreover, the knee was more flexed during AS compared to IS (R_KNEE_LT, AS—157.43°, IS—139.56°; L_KNEE_RT, AS—140.30°, IS—159.93°). In addition, greater flexion was determined in the hip joint (R_HIP_F_LT, AS—145.01°, IS—139.03°; L_HIP_F_RT, AS—138.33°, IS—145.39°). Based on those results, it can be stated that while performing a short turn a skier’s body is in a lower position with an emphasized side arc. These results are in line with those gathered from the kinetic analysis. Due to higher maximal forces on the outside leg, it is necessary to master the forces by gradually flexing the lower body segments and to adopt a body position with an emphasized hip abduction of the outside leg. By maintaining the mentioned side arc throughout the turn, it is possible to maintain the high centrifugal force without skidding and sliding.

Novel research focused on the kinematic analysis of recreational-level IS is scarce. Even more limited are studies concentrating on the specific implementation of IS as an additional activity for AS technique development.

One of the rare studies that was focused on a comparison of AS and IS was conducted by Kroll et al. [11]. The authors concluded that the angle of the body’s leaning towards the center of the turn is smaller during IS compared to AS. This can be explained by differences in the contact area between roller skates with the terrain and skis with the terrain. As already mentioned, the contact is more lateral during IS, which leads to a smaller side arc that a person needs to take in order to avoid skidding. Our research also confirms the previous statement, as we observed greater hip abduction (side arc) during AS. Moreover, the kinematic results in our study also support this kinetic analysis. In addition, research conducted by Kroll et al. [28] found that the greater knee flexion enables the skier to generate a higher force and to transfer pressure to the new outside leg more efficiently. The steepness of the terrain affects the skier’s body position, i.e., the side arc and the angles of lower body joints. Since AS is executed on a steeper terrain, the skier is in a lower position with a greater flexion of the knee and hip joint, which was confirmed in our study. Optimal flexion for a specific terrain and aligned lower body segments are a precondition for optimal pressure distribution. In our research, the pressure distribution was similar between AS and IS because of the similarity in body position that is necessary to execute a turn. However, the maximal pressure was higher in AS, meaning that a greater knee and hip flexion was required. Therefore, if the body position is optimal (in accordance with the demands of the terrain) the pressure distribution will be similar regardless of the maximal forces that occur.

Even though we determined some clear differences in kinematic aspects of the turn performance between IS and AS, based on our and some similar studies it can be concluded that the mechanism of turn execution is similar [17,29,30,31]. The skier has to maintain a lateral and frontal balance at the same time, which results in similar pressure distributions in IS and AS. Moreover, in both activities it is necessary to bring the center of mass low in relation to the inside of the turn radius. However, because of the specific conditions in which AS is performed, the skier has to be in a lower position in order to overcome higher foot pressure forces and master GRF when executing a turn. It is not possible to apply the same slope steepness and the corridor width when measuring IS and AS due to objective general differences between the mentioned activities. In the case of an identical slope steepness between IS and AS, the skier would not be able to control the speed during IS turns. This could cause compensatory movements and the executed turns would evidently differ from turns executed on skis. Therefore, the measured values would not be valid for comparison. In addition, the injury risk would be significantly higher while executing IS on terrain that was as steep as the ski slope. Another factor that would disable the control of the speed, and which would lead to inability to execute the turn in an identically set corridor, is the difference in the friction of the wheels compared to the skis with asphalt and snow, respectively. Moreover, wheels are exactly below the center of the foot, unlike ski edges, which are lateral in relation to the foot. Therefore, the lever is bigger on the roller skates and, in order to make a turn in the exact same direction, a smaller movement of the leg is required when executing a turn in roller skates. Considering proportionate adjustments to the terrain steepness and the length of the skis and roller skates, a narrower width of the corridor would be more appropriate for IS than for AS measurement. In that case, the subject executing the turn would have to apply almost the same movement amplitude on roller skates as they would on skis to achieve the same length, duration, rhythm and speed of the turn. The steepness of the terrain would be proportionally adjusted in order for the subject to achieve the same above-mentioned turn features with the same amplitude of joint movements. All of the above explains the reasons for choosing an AS slope with an inclination of 20° and a slope for IS with an inclination of 10°. If the AS slope steepness had been set to the same angle as in the IS measurement (10°), turns would not have been executed optimally compared to the ski slope regarding duration, turn, rhythm and speed. The same can be stated vice versa for a higher inclination of the IS slope. The observed additional activity (IS) can be used in training to simulate the conditions of the main activity (AS) with consideration of the already mentioned differences regarding terrain and equipment.

When observing IS as an additional activity for AS competitors, IS is also performed on less steep slopes. In such a way, the speed and the direction can be controlled while the rhythm and the tempo are similar to those achieved during AS. In our research, the focus was on recreational skiers whose level of skiing technique was not as high as that of skiing competitors. From that standpoint, it is even more necessary to adapt the IS training to the conditions and environments that are appropriate for recreational skiers. This enables them to perform IS training by themselves on available slopes in parks or other environments. Regarding training and developing ski technique, based on the above mentioned it is recommended to use IS as an additional activity in the preparation period of the season. During that period, alpine skiers train simpler course settings and elements. Turns are basic left–right with the same gate distance and gate off-set, while the courses are usually set on a flat terrain. Therefore, inline skating with its already mentioned characteristics could be used as an extra technical training with similar goals. When discussing recreational level alpine skiing, IS can provide the greatest benefits when used in periods when recreational level alpine skiers are in the first phase of short turn adoption. During that phase of the learning process, it is usually executed on a less steep terrain. In that case the forces are smaller and it is more suitable for the learning process of a short turn. Information about the timing of specific body movements that ensure the optimal control of the direction and speed could be crucial for accomplishing the aim of alpine ski schools—to enable skiers to be independent in mastering complex skiing elements. The obtained similarities and differences in some of the observed parameters of AS and IS can contribute to the development of the teaching process of each skiing technique. Since the results indicate similarities in pressure distribution when comparing AS and IS, the implementation of IS can help in mastering the pressure transfer in each phase of the turn. In addition, the coordination of movements and the sequence of lower limb actions are very similar in both activities. However, it has to be emphasized that the final values of kinematic parameters are slightly different due to the terrain and equipment, so the execution of the turn cannot be the same. Considering the mentioned differences, it is important to include IS as an additional activity only when recreational skiers have mastered the basics of AS turn performance. Only then can these skiers recognize the real contribution of IS in the learning process of pressure transfer and distribution. If the above-mentioned condition is not fulfilled, the skiers may aim to excessively simulate the movements during IS, which could maximize the potential injury risks and interfere with the learning process of AS. IS could also be used to master the rhythm for easier and smoother turn performance and for the timely performance of each movement, from the initial to the final phase of the turn. One of the problems that can be solved by using IS in the preparation period is oversteering. Oversteering can lead to a reduced control of the direction and an impaired dynamic balance, which can cause falls and injuries on the ski slope. IS helps in gaining a feel for steering, which prevents the skis turning faster than they do in their “natural” radius. Severe knee injuries can be caused if the skis turn faster due to an excessive edging angle that is not appropriate to the slope steepness and gate offset. Therefore, and as already mentioned, due to the different position of wheels in relation to the foot in IS, a bigger lever is created for the same knee movement compared to the AS. Hence, IS could be a useful tool in learning the optimal amplitude for executing turns and consequently prevent serious injuries.

### Limitations

The presented study was focused on the kinematic and kinetic analysis of the role of the outside leg when performing short parallel turns in AS and IS. In order to come to a clear conclusion whether it is justified to use IS as an additional activity for recreational alpine skiers, it is necessary to conduct an overall biomechanical analysis of the whole-body movement, including both the inside and outside leg and trunk movements. In addition, an electromyography analysis of the lower body’s muscle activity should be conducted to get an insight into the sequence of movements in each phase of the turn. Furthermore, to make an objective comparison of the two activities, it is necessary to completely replicate the conditions and the protocol in which the measurements are conducted. The corridor width and steepness of the slope differ due to general differences in the equipment and technical structure of the turn performance of each activity. Usually, training sessions in IS are performed in accordance with a temporal and geometrical extension of AS turns. However, due to abovementioned characteristics of both activities, the steepness of the terrain and corridor width cannot be exactly the same for both activities. In this way, the risk of potential injuries caused by an inadequate terrain steepness can be minimized.

## 5. Conclusions

The results of the conducted kinematic and kinetic analysis of the role of the outside leg in AS and IS pointed to clear similarities in the ratio of pressure distribution while performing short parallel turns. Furthermore, one of the main findings is determined differences in the kinematic parameters of the outside leg between AS and IS. The kinematic analysis pointed to differences in the knee and hip angle of the outside leg, which has a role in controlling the speed and direction during the descent. The kinematic and kinetic analyses were in accordance with each other. The maximal pressure was higher during AS, and it was to be expected that greater knee and hip flexion and hip abduction were to be found in AS. On the other hand, the pressure distribution was similar in AS and IS, meaning that the outside leg had the role of maintaining a dynamic balance and stability during short turn execution in both activities.

Although the motor knowledge necessary for the adoption of AS and IS is similar, it is important to be aware that there are objective differences which need to be taken into consideration when using IS as an additional activity for recreational skiers. IS can be used for solving more complex problems of skiing technique and adopting some specific high-level motor skills. Therefore, we recommend IS as an additional activity for skiers who have developed at least a basic AS technique in order to avoid interference with the learning process.

## Figures and Tables

**Table 1:** Results of MANOVA for short turns executed in IS and AS.

Test	Lambda Value	F	*p*
Wilks	0.05	169.1	0.00 *

Legend: * *p* < 0.05.

**Table 2:** Basic descriptive statistical parameters and MANOVA of kinematic parameters for IS and AS.

Variable	IS Mean ± SD	AS Mean ± SD	F	*p*
R_KNEE_LT (°)	157.43 ± 6.47	139.56 ± 2.07	483.64	0.00 *
R_HIP_F_LT (°)	145.01 ± 6.49	139.03 ± 5.84	32.82	0.00 *
R_HIP_AB_LT (°)	170.73 ± 2.24	163.68 ± 2.18	354.73	0.00 *
L_KNEE_RT (°)	159.93 ± 5.42	140.30 ± 2.95	142.87	0.00 *
L_HIP_F_RT (°)	145.39 ± 6.53	138.33 ± 9.35	20.37	0.00 *
L_HIP_AB_RT (°)	170.51 ± 1.25	163.00 ± 1.48	707.74	0.00 *

Legend: * *p* < 0.05; R_KNEE_LT—angle of the right knee flexion in the left turn; R_HIP_F_LT—angle of the right hip flexion in left turn; R_HIP_AB_LT—angle of the right hip abduction in the left turn; L_KNEE_RT—angle of the left knee flexion in the right turn; L_HIP_F_RT—angle of the left hip flexion in the right turn; L_HIP_AB_RT—angle of the left hip abduction in the right turn; (°)—degrees.

**Table 3:** Basic descriptive statistical parameters and MANOVA of kinetic parameters for IS and AS.

Variable	IS Mean ± SD	IS MBW **	AS Mean ± SD	AS MBW **	F	*p*
Max_R_LT (N)	584.82 ± 189.74	0.97	764.79 ± 176.24	1.27	33.81	0.00 *
Lat_R_LT (N)	34.36 ± 31.30	0.06	69.33 ± 86.72	0.12	10.07	0.00 *
Med_R_LT (N)	53.54 ± 61.73	0.09	159.64 ± 131.90	0.27	37.15	0.00 *
He_R_LT (N)	497.82 ± 155.80	0.83	535.82 ± 211.14	0.89	1.47	0.23
Max_L_RT (N)	564.44 ± 160.52	0.94	811.37 ± 124.28	1.35	103.57	0.00 *
Lat_L_RT (N)	38.62 ± 39.91	0.06	77.32 ± 93.98	0.13	10.05	0.00 *
Med_L_RT (N)	65.33 ± 69.87	0.11	161.33 ± 128.84	0.27	30.03	0.00 *
He__L_RT (N)	455.00 ± 130.74	0.76	554.47 ± 207.10	0.92	11.55	0.00 *

Legend: * *p* < 0.05; **—MBW = pressure (kg)/mean body weight of all participants (kg); Max_R_LT—maximum force of the right foot in the left turn; Lat_R_LT—force of the lateral side of the right foot in the left turn; Med_R_LT—force of the medial side of the right foot in the left turn; He_R_LT—force of the right heel in the left turn; Max_L_RT—maximum force of the left foot in the right turn; Lat_L_RT—force of the lateral side of the left foot in the right turn; Med_L_RT—force of the medial side of the left foot in the right turn; He__L_RT—force of the left heel in the right turn; (N)—newtons.

## Data Availability

The data presented in this study are available on request from the corresponding author. The data set is not publicly available due to its huge size and participants’ privacy protection.
